# Rare case of early neonatal sepsis caused by *Candida krusei* successfully treated with voriconazole

**DOI:** 10.1016/j.mmcr.2024.100659

**Published:** 2024-07-09

**Authors:** Shashi Bhushan, Supriya Mahajan, Aditya Sen

**Affiliations:** a Department of Paediatrics, School of Medical Sciences and Research, Sharda University, Greater Noida, UP, India; b Department of Microbiology, School of Medical Sciences and Research, Sharda University, Greater Noida, UP, India

**Keywords:** Neonatal sepsis, full term, *Candida krusei*, Voriconazole

## Abstract

This study reports a case of multidrug resistant *Candida krusei* as the cause of early neonatal sepsis in a term small-for-gestational age neonate weighing 1680 g that successfully responded to voriconazole therapy. Both blood culture and urine culture of the neonate sent on day 4 and day 8 respectively showed Gram positive oval budding yeast cells on Gram staining which was confirmed as *C. krusei* susceptible only to voriconazole by Vitek 2 Compact (Biomérieux, France) automated system. Voriconazole was given for fourteen days leading to good clinical response with microbiological clearance of fungus from blood and no side-effects.

## 1 Introduction

Invasive fungal infections (IFIs) in neonatal intensive care units (NICUs) are the second most common cause of infection-related death among critically ill neonates [1,2]. High-risk neonates become colonized with *Candida* spp. either vertically during vaginal birth, or horizontally from colonized hospital-workers during their stay in the NICU. Although *Candida albicans* remains the most common isolate in NICU, a shift to infections caused by non*-albicans Candida* (NAC) spp. *(C. parapsilosis, C. tropicalis, C. glabrata,* and *C. krusei*) has occurred during the last few decades [3].

Early onset neonatal invasive candidiasis (NIC) is considered to be a rare entity with publications limited to case reports or series that mainly highlight the milder noninvasive cutaneous variant. Although reports on invasive form of disease with early onset are uncommon, they generally describe a fulminant course of systemic illness, especially in extremely low birth weight (ELBW) infants (<1000 g) [4]. In contrast to early onset, *Candida* spp represents the third most common causative agent of late-onset sepsis with a high burden of morbidity and mortality [5].

Diagnosis of NIC is often difficult and challenging as clinical signs and symptoms are non-specific. Also, routine blood culture often do not report fungal organisms and therefore diagnosis requires a high index of suspicion. Even with high suspicion, blood cultures are often negative in neonatal fungal sepsis [6]. In recent times, there have been increasing reports of rarer species such as *C. krusei*, which have been recognised as a potentially multidrug resistant fungal pathogen due to its intrinsic fluconazole resistance and reports of decreased susceptibility to both flucytosine and amphotericin B [7]. There are only five articles in the medical literature that report on six isolated cases and an outbreak of seven cases of fungemia caused by *C. krusei* during the neonatal period all of which responded to liposomal amphotericin B and/or fluconazole. Of these, very few cases of early neonatal sepsis by *C. krusei* were reported [7].

The aim of this study is to report a case of neonatal fungal sepsis by multidrug resistant *C. krusei* in a term small-for-gestational age (SGA) neonate weighing 1680 g admitted in NICU and successfully treated with voriconazole.

## 2.Case presentation

A term SGA female neonate was born via emergency caesarean section with fetal distress to a primigravida mother with history of leaking per-vagina (PV) for more than 48 hrs. Mother registered for antenatal care at a tertiary care hospital with no history of hypertension or diabetes mellitus or any steroid intake before or during the pregnancy. Her first and second trimesters were uneventful. During the third trimester, she developed low grade intermittent fever and leaking PV 2–3 days before delivery. Her vaginal swab did not show evidence of *Candida* colonization. Her blood culture was sterile.

The neonate was low birth weight (LBW) weighing 1680g (day 0) with Apgar score of 5/10 at 1 minute and 6/10 at 5 minutes; hence post-resuscitation care was given with positive pressure ventilation (PPV) and subsequently she was transferred to the NICU. On day 1, the neonate developed intractable hypoglycemia requiring insertion of an umbilical venous catheter and glucose infusion, symptomatic polycythemia, abdominal distension, bilious regurgitation and neonatal seizures, with clinical suspicion of early onset sepsis. Cefotaxime (50 mg/kg/dose 12 hourly) and Amikacin (15 mg/kg/dose 24 hourly) were empirically started on the same day. On day 2, the neonate was shifted to Continuous Positive Airway Pressure (CPAP) in view of respiratory distress. On day 3, antibiotics were upgraded to piperacillin-tazobactam (100 mg/kg/dose IV 12 hourly), meropenem (20 mg/kg/dose 8 hourly IV) and vancomycin (15 mg/kg/dose 12 hourly). On day 4, despite broadspectrum antibiotics, therapeutic response was inadequate and fungal sepsis was suspected. Blood culture and urine samples were sent to detect any fungal pathogen as the cause of the neonatal sepsis.

**Fig. 1:**
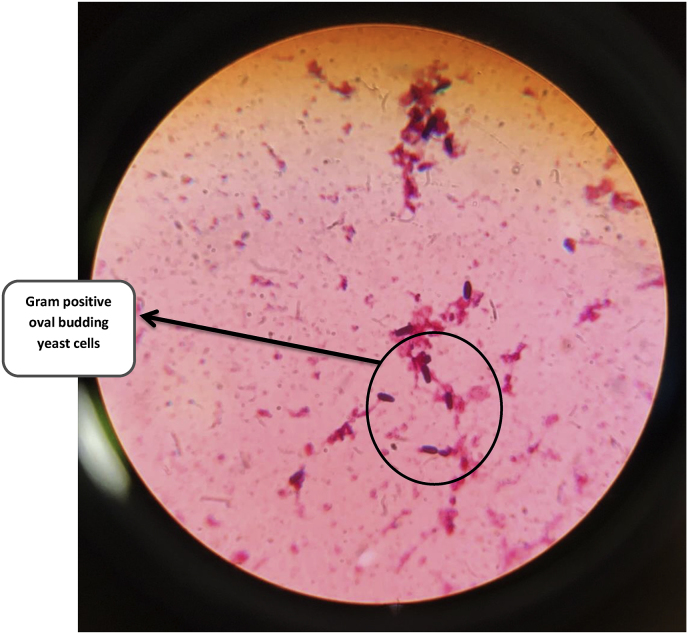
**(Coloured)** Direct Gram staining from positive blood culture bottle showing Gram positive oval budding yeast cells.

**Fig. 2:**
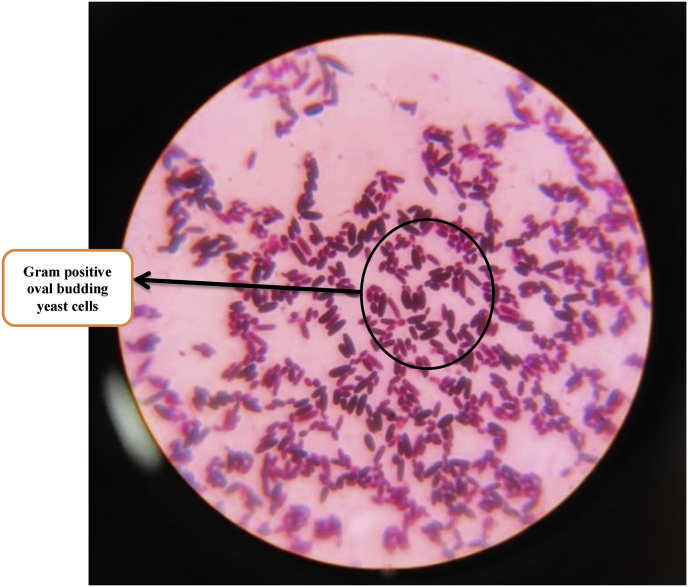
**(Coloured)** Gram staining done from colonies of SDA showing Gram positive oval budding yeast cells.

Urine microscopy revealed budding yeast cells for which fluconazole (20 mg/kg loading dose followed by 10 mg/kg per dose IV 24 hourly) was initiated on day 4. She also developed one episode of seizure for which Phenobarbitone (20 mg/kg IV loading dose followed by 4 mg/kg/day) was added. As seizures were not controlled, Levitiracetam (40 mg/kg/day loading dose followed by 20 mg/kg/day 12 hourly) was added. A lumbar puncture was performed and CSF cytology revealed increased WBCs, increased protein (450 mg/dl) and normal glucose. CSF Gram stain revealed few white blood cells and no microorganism. CSF culture was negative. Blood culture sent on day 4 flashed positive via BacT/Alert® system after two days of incubation (day 6). A direct Gram stain from the blood culture bottle revealed Gram positive oval budding yeast cells (and no bacterial elements) as shown in Fig. 1. Subculture was done from the blood culture bottle on Sabouraud Dextrose Agar (SDA), blood agar and MacConkey agar. On day 7, SDA showed growth of white, smooth, pasty and creamy colonies after incubation at 37 °C for 24 hrs and blood agar showed small, white, raised colonies after incubation at 37 °C for 24 hrs. MacConkey agar showed no growth. Gram staining done from the colonies of both SDA and blood agar also showed Gram positive oval budding yeast cells as shown in Fig. 2. Finally on day 8, *C. krusei* was detected as the cause of sepsis via Vitek 2 Compact (Biomérieux, France) automated system which was susceptible to Voriconazole (VCZ) (Minimum inhibitory concentration; MIC ≤ 0.12); intermediate to Caspofungin and Micafungin (MIC = 0.5) and resistant to Fluconazole (MIC = 4), Amphotericin B (MIC = 2) and Flucytosine (MIC = 16). Repeat urine sample was ordered for urine culture on day 8 which after 24 hrs of incubation revealed white, smooth, pasty and creamy colonies on SDA that also turned out to be *C. krusei* on Vitek 2 Compact (Biomérieux, France) automated system; depicting a probable dissemination of *C. krusei* in the urinary tract. Ultrasonography of abdomen revealed no evidence of fungal ball in the kidneys. Echocardiography demonstrated no evidence of any thrombosis or infective endocarditis. Fundoscopy showed no features of ocular candidiasis.

Fluconazole was stopped and VCZ was started on day 8 given by intravenous route as a loading dose of 6 mg/kg every 12 h on the first day of therapy, followed by 4 mg/kg every 12 h IV. IV VCZ was given for 8 days and then switched to oral and given for a total of 14 days after which the neonate started showing significant clinical improvement. TDM for VCZ could not be done due to lack of facilities in the institute.

Liver function tests (LFTs) at baseline (ALT = 15 IU/L and AST = 21 IU/L) and on day 7 (ALT = 11 IU/L and AST = 14 IU/L) of VCZ treatment were within normal limits. Blood culture resent after 7 days of starting VCZ therapy (day 15) revealed no fungal or bacterial growth. VCZ was given for a total of 14 days (day 22) and the neonate was discharged on day 23 owing to good clinical response with negative blood culture report and normal LFT.

## 3 Discussion

We report a case of *C. krusei* as the cause of early neonatal sepsis in a term SGA neonate. The unique feature of this case is the early onset of the NIC, a multidrug resistant *C. krusei* as the causative species, and the occurrence of NIC in a term neonate with a birth weight >1500 gr. Risk factors such as hypoglycemia, the presence of an umbilical venous catheter, and being SGA, might have contributed to the development of NIC.

Incidence of NIC is inversely related to birth weight. It has been documented that neonates with a birth weight of >1500 g have a low (0.06 %) incidence of NIC [8]. Although, Cook et al. showed that in low and middle income countries, 37 % neonates with NIC weighed >1500 g which contrasted dramatically with the already existing data (form high income countries) where extreme prematurity and extreme LBW neonates are the main high-risk groups for NIC [9].

Only few neonatal sepsis cases can be found in the literature in which *C. krusei* was the causative fungus [7,10]. The largest study to date (EUROCANDY), involving 23 pediatric centres demonstrated only 0.7 % cases to be caused by *C. krusei* [3]. A study by Amaral-Lopes et al. reported two cases of early neonatal sepsis and one case of late neonatal sepsis caused by *C. krusei*; all three of which responded favourably to liposomal amphotericin B [7].

The largest multi-country NeoOBS cohort study of NIC by Cook et al. included 127 infants with candidemia from 14 hospitals but *C. krusei* was not isolated in any of the cases [9].

Diagnosis and management of *NAC species* is challenging because signs and symptoms of fungal sepsis mimic bacterial sepsis and requires high degree of suspicion. Management of *NAC species* in particular is even more challenging due to their reduced susceptibility to fluconazole and varying susceptibility to other antifungal drugs necessitating routine antifungal susceptibility testing [11]. This condition remains a paradox because current Infectious Diseases Society of America guidelines recommend amphotericin B deoxycholate and fluconazole first-line therapies in neonatal patients with invasive candidiasis [12]. In this study also, the neonate was initially treated with fluconazole when urine microscopy revealed budding yeast cells to which she was not responding well and was shifted to VCZ when multidrug-resistant *C. krusei* was isolated on blood culture.

There is limited neonatal literature on VCZ use, treatment response, and adverse effects. In this study, the neonate responded well to treatment with VCZ showing both clinical resolution of symptoms and microbiological clearing of fungus with normal LFTs and no side effects. One other case report has reported the successful treatment of neonatal candidemia caused by *C. parapsilosis* with VCZ and no serious adverse reactions [13]. Although VCZ has not yet been approved for use in neonates, a recent study by Gastine et al. describes its use as empirical therapy in cases where no alternative is available [14]. Safety and efficacy of VCZ in infants has been clarified in another study where oral and intravenous formulations of VCZ was safely and effectively used to treat *Candida* CNS infection in preterm infants with no drug-related side effects [15]. Although there is no standard dosage guidelines of VCZ in neonates and infants, a Chinese single-center study pointed out that for most Asian children aged under 2 years, 5–7 mg/kg/dose twice daily is recommended for initial intravenous treatment. This study recommended this particular dosage on the basis of TDM performed using a validated in-house liquid chromatography–tandem mass spectrometry where it was observed that 5 to <7 mg/kg maintenance i. v. dose of VCZ significantly increased the number of children who reached therapeutic levels compared with lower dosages and dosages ≥7 mg/kg appeared to bring some additional benefit without statistical significance [16]. In our study, considering the non-availability of TDM facilities in our institute and considering the fact that VCZ was the only life-saving drug in this situation; the recommended VCZ dosage as per the above studies was given to the child.

In conclusion, VCZ appears to be a safe and life-saving antifungal to be used in critically ill newborns especially in multidrug resistant candidemia cases. More studies on VCZ in newborns need to be conducted to guide treatment in neonatal fungal sepsis.

## Funding

No financial support was obtained for this case report.

## Conflict of interest

The authors have no conflict of interest to declare.

This research did not receive any specific grant from funding agencies in the public, commercial, or not-for-profit sectors.

## CRediT authorship contribution statement

**Shashi Bhushan:** Writing – review & editing, Project administration, Investigation, Conceptualization. **Supriya Mahajan:** Writing – review & editing, Writing – original draft, Validation, Supervision, Resources, Methodology, Formal analysis. **Aditya Sen:** Writing – review & editing, Data curation.
