# Supplementary material for: Rare case of early neonatal sepsis caused by Candida krusei successfully treated with voriconazole
Source: Med Mycol Case Rep. 2024 Jul 9;45:100659. doi: 10.1016/j.mmcr.2024.100659 (PMC11301222; doi:10.1016/j.mmcr.2024.100659)
Supplement: Multimedia component 1 [file mmc1.pdf]

Medical Mycology Case Reports
ETHICAL FORM

Manuscript number (if applicable):	Author name: Dr. Supriya Mahajan

Medical Mycology Case Reports requires full disclosure of all sources of funding and potential conflicts of interest. The journal also requires a declaration that the author(s) have obtained written and signed consent to publish the case report from the patient or legal guardian(s).

If you have nothing to declare in any of these categories then this should be stated.

Funding Source

All sources of funding should be acknowledged and you should declare any extra funding you have received for academic research of this work. If there are none state 'there are none'.

Please state any sources of funding for your research

There are none

Conflict of Interest
Please declare any financial or personal interests that might be potentially viewed to influence the work presented. Interests could include consultancies, honoraria, patent ownership or other. If there are none state 'there are none'.

Please state any competing interests

There are none

Consent
Please declare that you have obtained written and signed consent to publish the case report from the patient or legal guardian(s).

Please state that consent has been obtained from the patient or legal guardian(s)

Written informed consent was obtained from the patient or legal guardian(s) for publication of this case report and accompanying images. A copy of the written consent is available for review by the Editor-in-Chief of this journal on request.

As corresponding author, I hereby declare that I sign this document on behalf of all the authors of the above mentioned manuscript.

Signature (a scanned signature is acceptable)

Supriya Mahajan.

Print name

Dr. Supriya Mahajan.
